# A comparison of ARMS and direct sequencing for *EGFR *mutation analysis and Tyrosine Kinase Inhibitors treatment prediction in body fluid samples of Non-Small-Cell Lung Cancer patients

**DOI:** 10.1186/1756-9966-30-111

**Published:** 2011-12-06

**Authors:** Yi Liu, Bing Liu, Xiao-Yan Li, Jian-Jie Li, Hai-Feng Qin, Chuan-Hao Tang, Wan-Feng Guo, Hai-Xu Hu, Sha Li, Cui-Jing Chen, Bing Liu, Hong-Jun Gao, Xiao-Qing Liu

**Affiliations:** 1 Cancer Center of People's Liberation Army of China, Affiliated Hospital of Academy of Military Medical Sciences, Beijing 100071, China

**Keywords:** Body Fluids, *EGFR *Mutation, Direct Sequencing, ARMS, TKIs, NSCLC

## Abstract

**Background:**

Epidermal growth factor receptor (*EGFR*) mutation is strongly associated with the therapeutic effect of tyrosine kinase inhibitors (TKIs) in patients with non-small-cell lung cancer (NSCLC). Nevertheless, tumor tissue that needed for mutation analysis is frequently unavailable. Body fluid was considered to be a feasible substitute for the analysis, but arising problems in clinical practice such as relatively lower mutation rate and poor clinical correlation are not yet fully resolved.

**Method:**

In this study, 50 patients (32 pleural fluids and 18 plasmas) with TKIs therapy experience and with direct sequencing results were selected from 220 patients for further analysis. The *EGFR *mutation status was re-evaluated by Amplification Refractory Mutation System (ARMS), and the clinical outcomes of TKIs were analyzed retrospectively.

**Results:**

As compared with direct sequencing, 16 positive and 23 negative patients were confirmed by ARMS, and the other 11 former negative patients (6 pleural fluids and 5 plasmas) were redefined as positive, with a fairly well clinical outcome (7 PR, 3 SD, and 1 PD). The objective response rate (ORR) of positive patients was significant, 81.3% (direct sequencing) and 72.7% (ARMS) for pleural fluids, and 80% (ARMS) for plasma. Notably, even reclassified by ARMS, the ORR for negative patients was still relatively high, 60% for pleural fluids and 46.2% for plasma.

**Conclusions:**

When using body fluids for *EGFR *mutation analysis, positive result is consistently a good indicator for TKIs therapy, and the predictive effect was no less than that of tumor tissue, no matter what method was employed. However, even reclassified by ARMS, the correlation between negative results and clinical outcome of TKIs was still unsatisfied. The results indicated that false negative mutation still existed, which may be settled by using method with sensitivity to single DNA molecule or by optimizing the extraction procedure with RNA or CTC to ensure adequate amount of tumor-derived nucleic acid for the test.

## Introduction

Lung cancer causes over 1 million deaths per year worldwide, making it the major source of cancer-related deaths [1].There has been progress made in therapeutic strategies for lung cancer, but the 5-year survival rate is still only about 15% [2]. Treatment strategies for lung cancer have changed dramatically with the recent discovery that a proportion of non-small cell lung cancers (NSCLC) harbor activating mutations in the epidermal growth factor receptor (*EGFR*) gene [3,4], and that the mutated *EGFR *proteins are particularly susceptible to inhibition by small-molecule tyrosine kinase inhibitors (TKIs) Gefitinib and Erlotinib [5-9].

In the 2011 Chinese edition of NCCN clinical practice guidelines of NSCLC, TKIs has been revised as first line therapy according to the latest randomized phase III studies such as IPASS, First-SIGNAL, WJTOG3405, OPTIMAL, and the presence of *EGFR*-activating mutation represents critical biological factor for proper patient selection [5-11]. As a result, *EGFR *mutations analysis has become a routine molecular test in many Chinese hospitals, and direct sequencing is the most frequently used method because it is readily available and relatively inexpensive to use as compared with assays of real-time PCR such as TaqMan probes, Amplification Refractory Mutation System (ARMS) and High Resolution Melting (HRM).

It is well known that the optimal DNA resource for *EGFR *mutation analysis is tumor tissue. Unfortunately, because most of the NSCLC patients were at the advanced stage and inoperable, sufficient tumor tissue was not readily available. For example, in IPASS study, only 36% (437/1217) of the patients had biopsied tissue suitable for testing, while in INTEREST study, the ratio is only 20% (297/1466) [5,12]. On the contrary, the sampling of body fluid such as pleural fluid and plasma is usually easy, less invasive, and repeatable, which are considered to be a feasible genomic DNA resources [13-18]. Nevertheless, the mutation test procedure using body fluids still needs to be optimized, standardized and validated.

In our hospital, patients who couldn't provide sufficient tumor tissues preferred to choose body fluids for EGFR mutation analysis, but two problems were found in our practice when direct sequencing was used. The first one was that the overall mutation rate was pretty lower than the average rate of Asian ethnic detected by sequencing (30-40%) [11], the second one was that quite a few patients response well with the TKIs therapy although their results of the mutation test are negative. We inferred that the low sensitivity of sequencing may result in the two problems. In order to verify this speculation, we selected 50 patients with TKIs therapy experience from the patients who joined the *EGFR *mutation analysis using body fluids, re-evaluated the *EGFR *mutation status of the extracted DNA by ARMS, a method with sensitivity of 1%, and analyzed the clinical outcome of TKIs retrospectively.

We found that ARMS could improve the mutation detection rate and the mutation positive patients responded well with TKIs therapy, but the correlation between mutation negative patients and TKIs therapy was still unsatisfactory. The results indicate that sensitivity of the method was not all the answers for the problems. We hypothesized that, as an alternative solution, the extraction procedure of nucleic acid should also be taken into consideration. The results of this study were reported in the present manuscript.

## Materials and methods

### Sample collection and processing

*EGFR *sequencing for exon 19 and 21 is one of the routine tests for NSCLC patients who want to initiate TKIs therapy in our hospital. The informed consent was obtained from each patient prior to the test. Pleural fluid samples were used as alternative clinical specimen for patients who couldn't provide sufficient tumor tissue. For patients who couldn't provide tumor tissue and pleural fluid, plasmas were used as an alternate. DNA was extracted from 400 μL supernatant of the pleural fluid or plasma by QIAamp DNA Blood Mini kit (Qiagen, Hilden, Germany) and eluted with 50 μL H_2_O. The extracted DNA was stored at -20°C until used.

*EGFR *exon 19 and 21 were amplified by polymerase chain reaction (PCR) using nested primer (Table 1) with Ex Taq polymerase (Takara, Tokyo, Japan). The first cycle of amplifications were performed using a 5 min initial denaturation at 95°C; followed by 30 cycles of 45 s at 95°C, 45 s at 54°C, and 1 min at 72°C; and a 6 min final extension at 72°C. Production of the first cycle was amplified in the secondary cycle using same condition as first one. The final products were cleared and sequenced with the internal primers using ABI PRISM 3730 DNA Analyser (Applied Biosystems, Foster City, CA, USA).

**Table 1:** the nested primer

	Sense	Antisense
19 exon outer	AAATAATCAGTGTGATTCGTGGAG	GAGGCCAGTGCTGTCTCTAAGG
19 exon inner	GTGCATCGCTGGTAACATCC	TGTGGAGATGAGCAGGGTCT
21 exon outer	GCAGCGGGTTACATCTTCTTTC	CAGCTCTGGCTCACACTACCAG
21 exon inner	GCTCAGAGCCTGGCATGAA	CATCCTCCCCTGCATGTGT

Patients were eligible for inclusion in the study for further analysis if they fit criteria as follow: ①With definite results of sequencing; ②The extracted DNA was of good quality and sufficient for extra test; ③With TKIs therapy experience and corresponding evaluation. The study was approved by the ethical committees of Affiliated Hospital of Academy of Military Medical Sciences.

The patients' DNA was re-tested by using ADx *EGFR *Mutations Detection Kit (Amoy Diagnostics, Xiamen, China), which has received State Food and Drug Administration (SFDA)'s approval for clinical usage in mainland China recently. The kit used the principle of Amplified Refractory Mutation System (ARMS) and covered the 29 *EGFR *mutation hotspots from exon 18 to 21. The assay was carried out according to the manufacturer's protocol with the MX3000P (Stratagene, La Jolla, USA) real-time PCR system. A positive or negative result could be reached if it met the criterion that was defined by the manufacturer's instruction. The results of ADx-AMRS were compared with those of direct sequencing.

### Treatment and evaluation

All the patients enrolled in the study had experience of TKIs therapy (Gefitinib or Erlotinib), although some of them were defined as mutation negative. The drugs were administered according to the manufacturer's instruction. TKIs therapy was not stopped until disease progression, unacceptable toxicity, or patient refusal happened (whichever was sooner). After the discontinuation of TKIs treatment, the patients were treated according to standard clinical practice at the discretion of the investigators.

Efficacy was assessed with computed tomography (CT) scans every 4 weeks until discontinuation or as clinically indicated. Responses were defined and categorized according to Response Evaluation Criteria in Solid Tumors (RECIST). All partial and complete responses were confirmed at least 4 weeks later with repeated imaging and a designation of stable disease required lack of progression for 8 weeks or more.

### Statistical analysis

Samples were examined to determine whether a statistically significant difference existed regarding variations in *EGFR *mutations between method of DNA sequencing and ADx-ARMS by the McNemar's test. The relationship between *EGFR *mutation and clinical outcome was examined by Fisher's exact test. Progression-free survivals (PFS) after TKIs therapy were analyzed by the Kaplan-Meier method, and were compared between groups by the log-rank test. The statistical analysis was carried out by using SAS software version 9.1.3 (SAS Institute, Inc., Cary, NC, USA).

## Results

### Characteristics of patients and samples

From December in 2008 to November in 2010, 220 patients joined the EGFR mutation analysis using body fluids since sufficient tumor tissues were unavailable after routine pathological examination was done. Among them, 142 were pleural fluids, and 78 were plasma. With direct sequencing, the corresponding mutation rate is 23.2% and 5.1% respectively (Table 2), lower than the average rate of Asian ethnic detected by sequencing on tumor tissues (30-40%) [11]. In addition, some mutation negative patients received TKIs therapy regardless the mutation status given the poor sensitivity of DNA sequencing and were found with good outcome (data not shown).

**Table 2:** Mutation rate for different kind of body fluid samples in our clinical practice using sequencing

	Pleural fluid	Plasma	Total
Total	142	78	220
19-del	18	2	20
L858R	15	2	17
Mutation rate (%)	23.2	5.1	16.8

We inferred that the low sensitivity of sequencing may result in the two problems. In order to verify this speculation, we tried to re-evaluate the EGFR mutation status of the extracted DNA by ARMS, a method with sensitivity of 1%. 50 patients were selected from the 220 patients according to the criteria mentioned in material and method part for further analysis. The samples included 32 pleural fluids and 18 plasmas. All the patients were Chinese and at the stage of IIIB or IV. The median age was 56.2 years (range, 31-77 years), and there were 32 males (64%) and 18 females (36%). The histological and/or cytological diagnosis for all the patients was adenocarcinoma. All the patients were treated with TKIs and evaluated for the response, 32 patients with Partial Response (PR), 7 with Stable Disease (SD), 11 with Progressive Disease (PD).

### *EGFR *mutation status and clinical outcome

The *EGFR *mutation status and clinical outcome for each patient was shown in Additional file 1. By direct sequencing, 16 samples were mutation positive and the other 34 were negative; By ADx-ARMS, 16 mutation positive and 23 negative samples were confirmed. However, 11 former negative samples (6 pleural fluids and 5 plasmas) were redefined as mutation positive. As shown in Table 3, for pleural fluid samples, ADx-ARMS was more sensitive than direct sequencing (χ^2 ^= 4.17 *P *= 0.0412). Nevertheless, the difference disappeared for plasma (Table 4, χ^2 ^= 3.2 *P *= 0.0736), which might be caused by small number of the samples.

**Table 3:** Statistics analysis for pleural fluid

ADx	Sequencing		Total
		
	+	-	
+	16	6	22
-	0	10	10
Total	16	16	32

χ^2 ^= 4.17 *P *= 0.0412

**Table 4:** Statistics analysis for Plasma

ADx	Sequencing		Total
		
	+	-	
+	0	5	5
-	0	13	13
Total	0	18	18

χ^2 ^= 3.2 *P *= 0.0736

**Figure 1:**
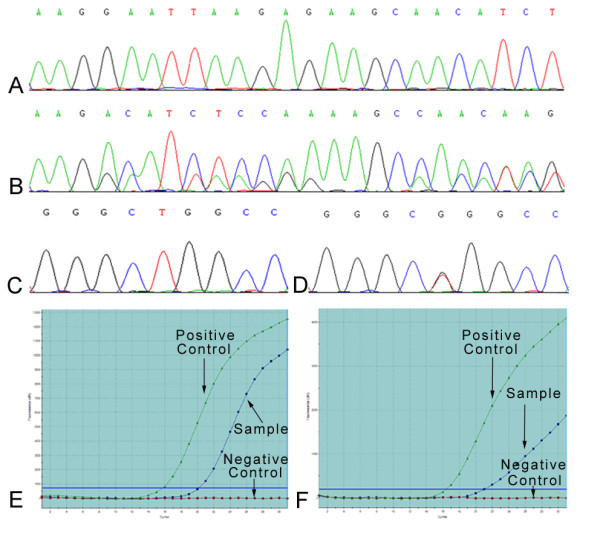
**Representative result for sequencing and ADx-ARMS**. A and E: No.36 patient 19 exon negative by sequencing but positive by ADx-ARMS. C and F: No.34 patient 21 exon negative by sequencing but positive by ADx-ARMS. B: No.13 patient 19 exon 746-751 del D: No.06 patient 21 exon L858R mutation

In addition, the ADx-ARMS identified 2 samples with both 19 del and L858R mutation, 4 with both 19 del and T790M mutation, and 1 with both L858R and L861Q or S768I (The two spots were designed in one tube, we could not differentiate it at that time). The representative results were showed in Figure 1.

### Comparison of the clinical evaluation

The comparison of the clinical evaluation was shown in Table 5. The therapeutic effect of TKIs was significant for the mutation positive patients. For pleural fluid samples, the objective response rate (ORR) of the 16 patients who were assessed as mutation positive by direct sequencing was 81.3% (13 PR, 2 SD, 1 PD), while the ORR of the 22 mutation positive patients detected by ADx-ARMS was 72.7% (16 PR, 5 SD, 1 PD), no difference was found between the two method (P = 0.706). For plasma samples, because none was defined as mutation positive by direct sequencing, the ORR was unavailable. However, regarding the 5 mutation positive patients redefined by ADx-ARMS, the ORR was 80% (4 PR, 1 PD). Although the ORR of mutation negative patients seemed lower than that of mutation positive one, statistical analysis showed no difference. For pleural fluid samples with direct sequencing used, the ORR for mutation positive and negative patients was 81.3% and 56.3%, respectively (P = 0.2524). For pleural fluids samples with ADx-ARMS used, the ORR for mutation positive and negative patients was 72.7% and 60%, respectively (P = 0.6828). For plasma samples with ADx-ARMS used, the ORR for mutation positive and negative patients was 80% and 46.2%, respectively (P = 0.3137). Even reclassified by a more sensitive method, the ORR for mutation negative patients was still relatively high, which was 60% for pleural fluid samples and 46.2% for plasma samples. Besides, as it was shown in Additional file 2, no difference was found in progression-free survival (PFS) among mutation positive and negative patients, no matter defined by sequencing or by ARMS. These results indicated that there might still be false negative mutations in these samples.

**Table 5:** Comparison of the clinical evaluation

		Pleural fluid		Plasma	
	
		Sequencing	ADx-ARMS	Sequencing	ADx-ARMS
Mutation positive	Number (%)	16(50%)	22(68.8%)	0	5(27.8%)
	PR	13	16	0	4
	SD	2	5	0	0
	PD	1	1	0	1
	ORR	81.3%^a^	72.7%^c^	NA	80%^e^

Mutation negative	Number (%)	16(50%)	10(31.2%)	18(100%)	13(72.2%)
	PR	9	6	10	6
	SD	4	1	1	1
	PD	3	3	7	6
	ORR	56.3%^b^	60%^d^	55.6%	46.2%^f^

PR = Partial Response; SD = Stable Disease; PD = Progressive Disease; ORR = Objective response rate

Between a and b, *P *= 0.2524; Between c and d, *P *= 0.6828; Between e and f, *P *= 0.3137; Between a and c, *P *= 0.706

## Discussion

Although it has been well recognized that *EGFR *mutation is strongly associated with the therapeutic effect of TKIs in NSCLC patients, most patients could not provide the tumor tissues that needed for the mutation test [5,12]. Prior literatures indicate that it is feasible to use the free DNA in body fluid such as pleural fluid and plasma as alternative clinical specimen for *EGFR *mutation analysis [13-18], but the procedure still needs to be optimized, standardized and validated.

The major finding of our research was that, when body fluid was used as substitute for *EGFR *mutation detection, the positive result was a good indicator for TKIs therapy, no matter it was detected by direct sequencing or ARMS. For patients who provided pleural fluid, the ORR of the 16 mutation positive patients defined by direct sequencing was 81.3%. When ARMS was used, 6 more patients were defined as mutation positive, with the ORR of the 22 patients at 72.7%. For patients who provided plasma, 5 mutation positive patients were detected only by ARMS, with the ORR at 80%. Generally, our result was consistent with that of OPTIMAL and IPASS research, both using tumor tissue for *EGFR *mutation analysis [5,9]. The ORR for mutation positive patients in OPTIMAL using direct sequencing was 83%, higher than that of IPASS using ARMS strategy (71.2%). Interestingly, such difference also occurred in our study using pleural fluid samples (81.3% Vs 72.7%). The results implied that, more sensitive methods such as ADx-ARMS may find more positive patients, but for them, mutative cells may represent a minority of the whole tumor, which may influence the final clinical outcome of TKIs. The explanation is consistent with the work of Qing Zhou et al. which found that the relative *EGFR *mutation abundance could predict benefit from *EGFR*-TKIs treatment for advanced NSCLC [19]. Our data emphasized that, for mutation positive results, the predictive effect of body fluid was no less than that of tumor tissue.

As considered for the two problems mentioned above, our research agreed with former reports that more sensitive method such as ARMS would be one of the feasible solutions [14,20]. Compared with direct sequencing, ADx-ARMS assay found 18.8% (6/32) and 27.8% (5/18) more patients to be mutation positive for pleural fluid and plasma, respectively. Direct sequencing is currently the routine method used to detect *EGFR *mutations. The merits of this method are readily available and economic, but the procedure is complicated and time-consuming. Meanwhile, the sensitivity of sequencing is about 30%, which tends to cause false negative result [21]. Given the poor sensitivity of DNA sequencing, many patients and physicians opt to start TKIs treatment even if the sequencing results were negative for *EGFR *mutation. If the tumor does not contain activating mutations on *EGFR*, treatment with TKIs will most likely be ineffective. In our study, 11 former negative patients (6 pleural fluids, 5 plasmas) defined by sequencing were proved to be positive at last, and the clinical outcome for them was quite satisfactory. If the treatment plan was made according to the result of direct sequencing, those patients may lose the chance of TKIs therapy.

Besides, by using ARMS, we also found 7 samples which harbouring double mutations (2 patients with 19 del and L858R, 1 with L858R and L861Q or S768I, 4 with 19 del and T790M). The clinical evaluations for the former 3 patients were all PR. This result was consistent with the study of Zhang et al. [22] which showed that patients with double activating mutations involving both exons 19 and 21 tend to respond well to TKIs and the sensitivity to TKIs was enhanced compared with either single mutant. As demonstrated by Qing Zhou et al. that the relative *EGFR *mutation abundance could predict benefit from *EGFR*-TKIs treatment [19], we hypothesized that the clinical benefits (3PR, 1SD) of the 4 patients which harbouring both 19 del and T790M may be owing to the dominant composition in 19 del.

Notably, even reclassified by ARMS, no difference was found in PFS among mutation positive and negative patients, the ORR for negative patients was still relatively high, 60% for pleural fluids and 46.2% for plasma, higher than that of IPASS (1.1%) and First-SIGNAL (25.9%) research [5,6]. Taking into consideration that all the patients in our research were adenocarcinoma, the well known type of lung cancer that can get maximum benefit from TKIs therapy, and the low abundance of DNA in body fluid, the results indicated that there might still be false negative mutations in these samples. We presumed that the phenomenon can be explained in two aspects.

Firstly, the sensitivity of ARMS is 1%, nevertheless, if the abundance of the mutation DNA was below this limitation, false negative results were inevitable. Prior literature indicated that, using ARMS for plasma samples, the false negative rate was still relatively high, which was about 30% as compared with tumor tissue [13,23]. Recently, Yung TK et al. reported a method named Microfluidics Digital PCR, which could detect a single-mutant DNA molecule and precisely determine the quantities of mutant and wild-type sequences. By using this method, the sensitivity and specificity of plasma *EGFR *mutation analysis reached 92% and 100% respectively, as compared with the sequencing results of tumor samples [18]. This method may be more suitable than ARMS for *EGFR *mutation analysis using body fluid samples, but it is not readily available now and more stringent clinical evidence is still needed in the future.

Secondly, regardless of the sensitivity of detection method, if tumor-derived DNA was not contained in the body fluid sample, the mutation analysis was obviously in vain.

For pleural fluid samples, it is well recognized that cell pellets could be used to ensure tumor cells was contained in the sample. Nevertheless, in a significant proportion of patients (30-40%), the yield of malignant cells from thoracentesis is inadequate for cytological and molecular diagnostic testing. We used cell-free pleural fluids in this study because it is abundant. Meanwhile, prior literature demonstrated that when sensitive genotyping assays was used, cell-free pleural fluid could provide the same mutational information as pleural effusion cells [15]. The problem is that, when cell-free pleural fluid was used, it was impossible to precisely evaluate whether the tumor-derived DNA was adequately contained, since the extracted free DNA arises not only from tumor cells, but also from the necrotic or apoptotic nontumor cells. Recently, free RNA in pleural fluid as a favouring material for *EGFR *mutation analysis was attracting more and more attention. The high *EGFR *mutation rate of free RNA in pleural effusion has been reported in the article by Wu et al. [24]. Later on, the same research group found out that the mutation-detection yield of sequencing from RNA was coupled with the superior prediction of clinical efficacy to first-line TKIs [25]. The explanation was that, contaminated nontumor cells within pleural fluid may have no or lower *EGFR *expression, using RNA instead of genomic DNA as the source for *EGFR *mutation analysis could minimize the influence of nontumor cells.

For blood samples, most reports used plasma rather than cell pellets for mutation analysis, because tumor cells in the blood are rare as compared with the cells of hematopoietic lineages. The documented sensitivity of plasma varied from 33% to 100%, which may be resulted from various detection methods or from different patients enrolled [17,18,23,26,27]. But using plasma encounter the same problem as using cell-free pleural fluid, namely, it is impossible to precisely evaluate whether the tumor-derived DNA was adequately contained. The characterization of circulating tumor cell might resolve the problem ultimately, since it is ascertain that the test was done on tumor cells. In the study by Maheswaran et al, there were 12 patients for whom specimens of the primary tumor, CTCs, and plasma were all available for *EGFR *mutation analysis. The genotyping of CTCs appeared to be more sensitive than plasma (92% Vs 33%, *P*= 0.009) [27]. The main problem now is that the technology of CTC enrichment still needs to be standardized and generalized. In recent years, tremendous efforts have been made on CTC detection and characterization [28,29]. In the near future, *EGFR *mutation analysis on CTC may become a reality in the routine clinical practice.

Our study had two limitations, which hindered us from verifying the hypothesis mentioned above. First, although we and others have demonstrated that body fluid is feasible [13-18], analysis for *EGFR *mutations with DNA extracted from tumor tissue remains the gold standard. Nevertheless, since all the patients enrolled in this study couldn't provide sufficient tumor tissue after routine pathological examination was done, the mutation status of the tumor tissue were not available and we could not testify whether there were still false negative results left after the extracted DNA were re-examined by ARMS. Second, although it is necessary to re-extract the nucleic acid with an optimized procedure by RNA or CTC, and then, to compare the mutation analysis with current study, the original body fluid samples of the patients were not preserved after the mutation analysis was done, the comparison could not be carried out. In order to address the two issues above, we had set a new research plan and the patients were now under enrolling.

## Conclusions

In conclusion, our finding suggested that, when body fluids were used for *EGFR *mutation analysis, positive result is consistently a good indicator for TKIs therapy, and the predictive effect was no less than that of tumor tissue, no matter what method was employed. Conversely, for negative result, we should be highly cautious due to its poor correlation with the response of TKIs therapy. The problem may be settled by using method with sensitivity to single DNA molecule such as Digital PCR or by optimizing the extraction procedure with RNA or CTC to ensure adequate amount of tumor-derived nucleic acid for the test.

## Competing interests

'The authors declare that they have no competing interests.

## Authors' contributions

YL carried out the molecular genetic studies and drafted the manuscript. BL*, HXH and SL carried out the molecular analysis. BL*, XYL, JJL, HFQ, CHT, WFG, CJC and HJG provide the body fluid samples and clinical data of the patients. YL, BL and XQL participated in the design and coordination of the study. All authors reviewed the draft manuscript and read and approved the final version for submission

## Supplementary Material

Additional file 1 ***EGFR *mutation status and clinical outcome for each patient**. The file contains the *EGFR *mutation status (detected by sequencing and ARMS) and the clinical outcome (evaluation and PFS) for each patient. Click here for file

Additional file 2 **Kaplan-Meier analysis for PFS**. The file contains Kaplan-Meier analysis for PFS in 3 categories of patients: pleural fluid samples using sequencing, pleural fluid samples using ARMS, plasma samples using ARMS. Click here for file

## References

[B1] Jemal A Siegel R Xu J Ward E Cancer Statistics, 2010 CA Cancer J Clin 2010 60 277 300 10.3322/caac.20073 20610543

[B2] American Cancer Society Cancer Facts & Figures 2010 2010 Atlanta: American Cancer Society

[B3] Paez JG Jänne PA Lee JC Tracy S Greulich H Gabriel S Herman P Kaye FJ Lindeman N Boggon TJ Naoki K Sasaki H Fujii Y Eck MJ Sellers WR Johnson BE Meyerson M *EGFR *mutations in lung cancer: correlation with clinical response to gefitinib therapy Science 2004 304 1497 1500 10.1126/science.1099314 15118125

[B4] Lynch TJ Bell DW Sordella R Gurubhagavatula S Okimoto RA Brannigan BW Harris PL Haserlat SM Supko JG Haluska FG Louis DN Christiani DC Settleman J Haber DA Activating mutations in the epidermal growth factor receptor underlying responsiveness of non-small-cell lung cancer to gefitinib N Engl J Med 2004 350 2129 2139 10.1056/NEJMoa040938 15118073

[B5] Mok TS Wu YL Thongprasert S Yang CH Chu DT Saijo N Sunpaweravong P Han B Margono B Ichinose Y Nishiwaki Y Ohe Y Yang JJ Chewaskulyong B Jiang H Duffield EL Watkins CL Armour AA Fukuoka M Gefitinib or carboplatin-paclitaxel in pulmonary adenocarcinoma N Engl J Med 2009 361 947 957 10.1056/NEJMoa0810699 19692680

[B6] Lee JS Park K Kim SW A randomized phase III study of gefitinib versus standard chemotherapy (gemcitabine plus cisplatin) as a first-line treatment for never smokers with advanced or metastatic adenocarcinoma of the lung13th World Conference on Lung Cancer, San Francisco 2009 (abstr PRS.4)

[B7] Maemondo M Inoue A Kobayashi K Sugawara S Oizumi S Isobe H Gemma A Harada M Yoshizawa H Kinoshita I Fujita Y Okinaga S Hirano H Yoshimori K Harada T Ogura T Ando M Miyazawa H Tanaka T Saijo Y Hagiwara K Morita S Nukiwa T North-East Japan Study Group Gefitinib or chemotherapy for non-small-cell lung cancer with mutated *EGFR* N Engl J Med 2010 362 2380 2388 10.1056/NEJMoa0909530 20573926

[B8] Mitsudomi T Morita S Yatabe Y Negoro S Okamoto I Tsurutani J Seto T Satouchi M Tada H Hirashima T Asami K Katakami N Takada M Yoshioka H Shibata K Kudoh S Shimizu E Saito H Toyooka S Nakagawa K Fukuoka M West Japan Oncology Group Gefitinib versus cisplatin plus docetaxel in patients with non-small-cell lung cancer harbouring mutations of the epidermal growth factor receptor (WJTOG3405): An open label, randomised phase 3 trial Lancet Oncol 2010 11 121 128 10.1016/S1470-2045(09)70364-X 20022809

[B9] Zhou C Wu YL Chen G Efficacy results from the randomised phase III OPTIMAL (CTONG 0802) study comparing first-line erlotinib versus carboplatin (CBDCA) plus gemcitabine (GEM), in Chinese advanced non-small-cell lung cancer (NSCLC) patients (PTS) with *EGFR *activating mutations Ann Oncol 2010 21 6 (suppl 8)

[B10] Keedy VL Temin S Somerfield MR Beasley MB Johnson DH McShane LM Milton DT Strawn JR Wakelee HA Giaccone G American Society of Clinical Oncology Provisional Clinical Opinion: Epidermal Growth Factor Receptor (*EGFR*) Mutation Testing for Patients With Advanced Non-Small-Cell Lung Cancer Considering First-Line *EGFR *Tyrosine Kinase Inhibitor Therapy J Clin Oncol 2011 29 2121 7 10.1200/JCO.2010.31.8923 21482992

[B11] The Chinese Edition of NCCN Clinical Practice Guidelines in Oncology-Non-Small Cell Lung Cancer Guideline 2011

[B12] Kim ES Hirsh V Mok T Socinski MA Gervais R Wu YL Li LY Watkins CL Sellers MV Lowe ES Sun Y Liao ML Osterlind K Reck M Armour AA Shepherd FA Lippman SM Douillard JY Gefitinib versus docetaxel in previously treated non-small-cell lung cancer (INTEREST): a randomised phase III trial The Lancet 2008 372 1809 1818 10.1016/S0140-6736(08)61758-4 19027483

[B13] Kimura H Suminoe M Kasahara K Sone T Araya T Tamori S Koizumi F Nishio K Miyamoto K Fujimura M Nakao S Evaluation of epidermal growth factor receptor mutation status in serum DNA as a predictor of response to gefitinib (IRESSA) Br J Cancer 2007 97 6 778 84 10.1038/sj.bjc.6603949 17848912 PMC2360394

[B14] Kimura H Fujiwara Y Sone T Kunitoh H Tamura T Kasahara K Nishio K High sensitivity detection of epidermal growth factor receptor mutations in the pleural effusion of non-small cell lung cancer patients Cancer Sci 2006 97 7 642 8 10.1111/j.1349-7006.2006.00216.x 16827805 PMC11160100

[B15] Zhang X Zhao Y Wang M Yap WS Chang AY Detection and comparison of epidermal growth factor receptor mutations in cells and fluid of malignant pleural effusion in non-small cell lung cancer Lung Cancer 2008 60 2 175 82 10.1016/j.lungcan.2007.10.011 18061305

[B16] Brevet M Johnson ML Azzoli CG Ladanyi M Detection of *EGFR *mutations in plasma DNA from lung cancer patients by mass spectrometry genotyping is predictive of tumor *EGFR *status and response to *EGFR *inhibitors Lung Cancer 2011 73 1 96 102 10.1016/j.lungcan.2010.10.014 21130517 PMC3282180

[B17] Bai H Mao L Wang HS Zhao J Yang L An TT Wang X Duan CJ Wu NM Guo ZQ Liu YX Liu HN Wang YY Wang J Epidermal growth factor receptor mutations in plasma DNA samples predict tumor response in Chinese patients with stages IIIB to IV non-small-cell lung cancer J Clin Oncol 2009 27 2653 9 10.1200/JCO.2008.17.3930 19414683

[B18] Yung TK Chan KC Mok TS Tong J To KF Lo YM Single-molecule detection of epidermal growth factor receptor mutations in plasma by microfluidics digital PCR in non-small cell lung cancer patients Clin Cancer Res 2009 15 6 2076 84 10.1158/1078-0432.CCR-08-2622 19276259

[B19] Zhou Q Zhang XC Chen ZH Yin XL Yang JJ Xu CR Yan HH Chen HJ Su J Zhong WZ Yang XN An SJ Wang BC Huang YS Wang Z Wu YL Relative Abundance of *EGFR *Mutations Predicts Benefit From Gefitinib Treatment for Advanced Non-Small-Cell Lung Cancer J Clin Oncol 2011 29 24 3316 3321 10.1200/JCO.2010.33.3757 21788562

[B20] Ellison G Donald E McWalter G Knight L Fletcher L Sherwood J Cantarini M Orr M Speake G A comparison of ARMS and DNA sequencing for mutation analysis in clinical biopsy samples J Exp Clin Cancer Res 2010 29 132 10.1186/1756-9966-29-132 20925915 PMC2988723

[B21] Fan X Furnari FB Cavenee WK Castresana JS Non-isotopic silver-stained SSCP is more sensitive than automated direct sequencing for the detection of PTEN mutations in a mixture of DNA extracted from normal and tumor cells Int J Oncol 2001 18 5 1023 6 11295051 10.3892/ijo.18.5.1023

[B22] Zhang GC Lin JY Wang Z Zhou Q Xu CR Zhu JQ Wang K Yang XN Chen G Yang JJ Huang YJ Liao RQ Wu YL Epidermal growth factor receptor double activating mutations involving both exons 19 and 21 exist in Chinese non-small cell lung cancer patients Clin Oncol (R Coll Radiol) 2007 19 7 499 506 10.1016/j.clon.2007.04.006 17537621

[B23] Kuang Y Rogers A Yeap BY Wang L Makrigiorgos M Vetrand K Thiede S Distel RJ Jänne PA Non- invasive detection of *EGFR *T790M in gefitinib or erlotinib resistant non-small cell lung cancer Clin Cancer Res 2009 15 2630 6 10.1158/1078-0432.CCR-08-2592 19351754 PMC2727796

[B24] Wu SG Gow CH Yu CJ Chang YL Yang CH Hsu YC Shih JY Lee YC Yang PC Frequent epidermal growth factor receptor gene mutations in malignant pleural effusion of lung adenocarcinoma Eur Respir J 2008 32 4 924 30 10.1183/09031936.00167407 18508816

[B25] Tsai TH Su KY Wu SG Chang YL Luo SC Jan IS Yu CJ Yu SL Shih JY Yang PC RNA is Favorable for Analyzing EGFR Mutations in Malignant Pleural Effusion of Lung Cancer Eur Respir J 2011 in press 10.1183/09031936.00043511 21719485

[B26] He C Liu M Zhou C Zhang J Ouyang M Zhong N Xu J Detection of epidermal growth factor receptor mutations in plasma by mutant-enriched PCR assay for prediction of the response to gefitinib in patients with non-small-cell lung cancer Int J Cancer 2009 125 2393 9 10.1002/ijc.24653 19530244

[B27] Maheswaran S Sequist LV Nagrath S Ulkus L Brannigan B Collura CV Inserra E Diederichs S Iafrate AJ Bell DW Digumarthy S Muzikansky A Irimia D Settleman J Tompkins RG Lynch TJ Toner M Haber DA Detection of mutations in *EGFR *in circulating lung-cancer cells N Engl J Med 2008 359 4 366 77 10.1056/NEJMoa0800668 18596266 PMC3551471

[B28] Pantel K Alix-Panabières C Circulating tumour cells in cancer patients: challenges and perspectives Trends Mol Med 2010 16 9 398 406 10.1016/j.molmed.2010.07.001 20667783

[B29] Alunni-Fabbroni M Sandri MT Circulating tumour cells in clinical practice: Methods of detection and possible characterization Methods 2010 50 4 289 97 10.1016/j.ymeth.2010.01.027 20116432

