# Supplementary material for: A comparison of ARMS and direct sequencing for EGFR mutation analysis and Tyrosine Kinase Inhibitors treatment prediction in body fluid samples of Non-Small-Cell Lung Cancer patients
Source: J Exp Clin Cancer Res. 2011 Dec 6;30(1):111. doi: 10.1186/1756-9966-30-111 (PMC3287118; doi:10.1186/1756-9966-30-111)
Supplement: Additional file 1 — EGFR mutation status and clinical outcome for each patient. The file contains the EGFR mutation status (detected by sequencing and ARMS) and the clinical outcome (evaluation and PFS) for each patient. [file 1756-9966-30-111-S1.DOC]

# Additional files

### Additional file 1 –*EGFR* mutation status and clinical outcome for each patients

| pleural fluids | | | | | | | |
| --- | --- | --- | --- | --- | --- | --- | --- |
| NO. | Seq | ADx-ARMS | Res | NO. | Seq | ADx-ARMS | Res |
| 1 | 19 del | 19 del | PR | 17 | Wild | L858R | SD |
| 2 | 19 del | 19 del | PD | 18 | Wild | 19 del and L858R | PR |
| 3 | 19 del | 19 del | PR | 19 | Wild | L858R | PR |
| 4 | 19 del | 19 del and T790M | PR | 20 | Wild | 19 del | SD |
| 5 | L858R | L858R and L861Q or S768I | PR | 21 | Wild | 19 del | SD |
| 6 | L858R | L858R | SD | 22 | Wild | 19 del | PR |
| 7 | 19 del | 19 del | PR | 23 | Wild | Wild | PD |
| 8 | 19 del | 19 del and T790M | SD | 24 | Wild | Wild | PR |
| 9 | 19 del | 19 del and T790M | PR | 25 | Wild | Wild | SD |
| 10 | 19 del | 19 del | PR | 26 | Wild | Wild | PR |
| 11 | 19 del | 19 del | PR | 27 | Wild | Wild | PR |
| 12 | L858R | 19 del and L858R | PR | 28 | Wild | Wild | PD |
| 13 | 19 del | 19 del | PR | 29 | Wild | Wild | PR |
| 14 | 19 del | 19 del and T790M | PR | 30 | Wild | Wild | PD |
| 15 | L858R | L858R | PR | 31 | Wild | Wild | PR |
| 16 | L858R | L858R | PR | 32 | Wild | Wild | PR |
| Plasma | | | | | | | |
| No. | Seq | ADx-AMRS | Res | No. | Seq | ADx-ARMS | Res |
| 33 | Wild | 19 del | PD | 42 | Wild | Wild | PD |
| 34 | Wild | L858R | PR | 43 | Wild | Wild | PR |
| 35 | Wild | 19 del | PR | 44 | Wild | Wild | PD |
| 36 | Wild | 19 del | PR | 45 | Wild | Wild | PR |
| 37 | Wild | 19 del | PR | 46 | Wild | Wild | PR |
| 38 | Wild | Wild | PR | 47 | Wild | Wild | PD |
| 39 | Wild | Wild | SD | 48 | Wild | Wild | PD |
| 40 | Wild | Wild | PD | 49 | Wild | Wild | PD |
| 41 | Wild | Wild | PR | 50 | Wild | Wild | PR |

PR=Partial response; SD=Stable disease; PD=Progressive disease; Seq=Sequencing; Res=Response
