# Supplementary material for: A comparison of ARMS and direct sequencing for EGFR mutation analysis and Tyrosine Kinase Inhibitors treatment prediction in body fluid samples of Non-Small-Cell Lung Cancer patients
Source: J Exp Clin Cancer Res. 2011 Dec 6;30(1):111. doi: 10.1186/1756-9966-30-111 (PMC3287118; doi:10.1186/1756-9966-30-111)
Supplement: Additional file 2 — Kaplan-Meier analysis for PFS. The file contains Kaplan-Meier analysis for PFS in 3 categories of patients: pleural fluid samples using sequencing, pleural fluid samples using ARMS, plasma samples using ARMS. [file 1756-9966-30-111-S2.DOC]

# Additional files

### Additional file 2 –Kaplan-Meier analysis forPFS


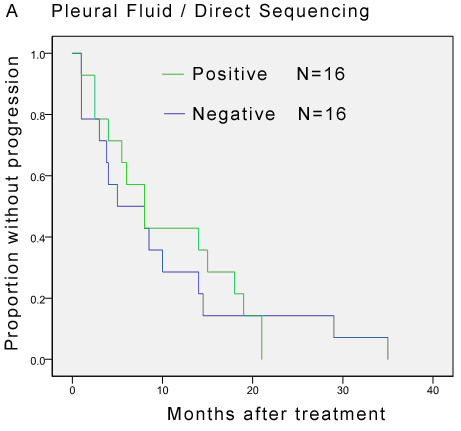


| sequencing | Mean^a^ | | | | Median | | | |
| --- | --- | --- | --- | --- | --- | --- | --- | --- |
|  | Estimate | Std. Error | 95% Confidence Interval | | Estimate | Std. Error | 95% Confidence Interval | |
|  |  |  | Lower Bound | Upper Bound |  |  | Lower Bound | Upper Bound |
| Negative | 9.843 | 2.793 | 4.369 | 15.317 | 5.000 | 3.742 | .000 | 12.334 |
| Positive | 10.393 | 1.961 | 6.550 | 14.236 | 8.000 | 1.852 | 4.371 | 11.629 |
| Overall | 10.118 | 1.675 | 6.835 | 13.401 | 8.000 | 1.637 | 4.792 | 11.208 |
| a. Estimation is limited to the largest survival time if it is censored. | | | | | | | | |

| **Overall Comparisons** | | | |
| --- | --- | --- | --- |
|  | Chi-Square | df | Sig. |
| Log Rank (Mantel-Cox) | .023 | 1 | .880 |


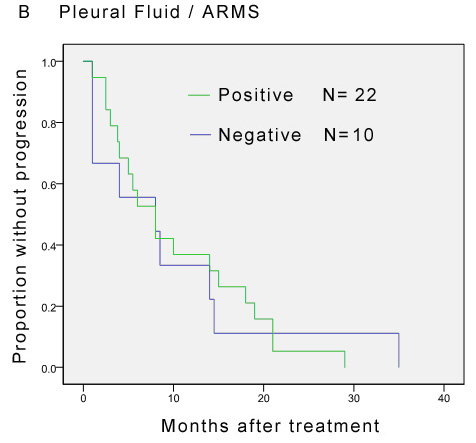


| **Means and Medians for Survival Time** | | | | | | | | |
| --- | --- | --- | --- | --- | --- | --- | --- | --- |
| ARMS | Mean^a^ | | | | Median | | | |
|  | Estimate | Std. Error | 95% Confidence Interval | | Estimate | Std. Error | 95% Confidence Interval | |
|  |  |  | Lower Bound | Upper Bound |  |  | Lower Bound | Upper Bound |
| Negative | 9.667 | 3.624 | 2.564 | 16.769 | 8.000 | 5.963 | .000 | 19.687 |
| Positive | 10.332 | 1.852 | 6.702 | 13.962 | 8.000 | 1.793 | 4.485 | 11.515 |
| Overall | 10.118 | 1.675 | 6.835 | 13.401 | 8.000 | 1.637 | 4.792 | 11.208 |
| a. Estimation is limited to the largest survival time if it is censored. | | | | | | | | |

| **Overall Comparisons** | | | |
| --- | --- | --- | --- |
|  | Chi-Square | df | Sig. |
| Log Rank (Mantel-Cox) | .008 | 1 | .927 |


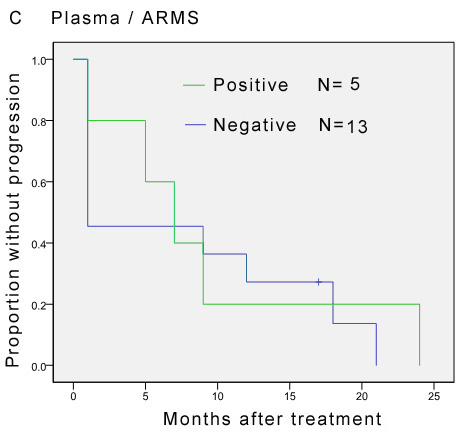


| **Means and Medians for Survival Time** | | | | | | | | |
| --- | --- | --- | --- | --- | --- | --- | --- | --- |
| ARMS | Mean^a^ | | | | Median | | | |
|  | Estimate | Std. Error | 95% Confidence Interval | | Estimate | Std. Error | 95% Confidence Interval | |
|  |  |  | Lower Bound | Upper Bound |  |  | Lower Bound | Upper Bound |
| Negative | 7.773 | 2.566 | 2.742 | 12.803 | 1.000 | . | . | . |
| Positive | 9.200 | 3.929 | 1.498 | 16.902 | 7.000 | 2.191 | 2.706 | 11.294 |
| Overall | 8.313 | 2.124 | 4.150 | 12.475 | 5.000 | 6.000 | .000 | 16.760 |
| a. Estimation is limited to the largest survival time if it is censored. | | | | | | | | |

| **Overall Comparisons** | | | |
| --- | --- | --- | --- |
|  | Chi-Square | df | Sig. |
| Log Rank (Mantel-Cox) | .159 | 1 | .690 |
